# A Novel MAX Gene Mutation Variant in a Patient With Multiple and “Composite” Neuroendocrine–Neuroblastic Tumors

**DOI:** 10.3389/fendo.2020.00234

**Published:** 2020-05-19

**Authors:** Carlotta Pozza, Franz Sesti, Carla Di Dato, Emilia Sbardella, Riccardo Pofi, Francesca Schiavi, Vincenzo Bonifacio, Andrea M. Isidori, Antongiulio Faggiano, Andrea Lenzi, Elisa Giannetta

**Affiliations:** ^1^Department of Experimental Medicine, Sapienza University of Rome, Rome, Italy; ^2^Familial Cancer Clinic and Oncoendocrinology, Veneto Institute of Oncology, IRCCS, Padua, Italy

**Keywords:** composite NEN, pheochromocytoma, ganglioneuroblastoma, *MAX* gene, paraganglioma

## Abstract

**Introduction:** Pheochromocytomas (PCCs), paragangliomas (PGLs), ganglioneuroblastomas (GNBs), and ganglioneuromas (GNs) are neuroendocrine neoplasms (NENs) that were thought to share a common embryologic origin from neural crest cells. However, they rarely occur concurrently and recurrently. We describe the case of a 40-years-old woman with “composite PCC-GN” and multiple NENs and neuroblastic tumors.

**Case presentation:** The patient was first referred to our department at the age of 15 years for paroxysmal hypertension, headache, sweating, and watery diarrhea. Her personal history included the diagnosis of a pelvic GNB with lumbar–aortic lymph node metastases at 11 months. Her family history was positive for cerebral glioblastoma multiforme (father). An abdominal ultrasound showed a right adrenal mass that histologically was a “composite adrenal PCC-GN.” The symptoms disappeared after surgery. At the age of 20 years, the symptoms returned: computed tomography (CT) and 131I-metaiodobenzylguanidine (MIBG) scintigraphy showed an inter-aortocaval mass, found histologically to be an inter-aortocaval PGL. Her symptoms reappeared again at 28 years: CT and magnetic resonance imaging revealed four left adrenal gland nodules, found histologically to be multifocal PCCs with some atypia. Genetic screening for *VHL, RET, NF1, Tp53, SDHD, SDHB, SDHC, SDHAF2, SDHAF3, SDHA*, and *TMEM127* was negative. Mutational analysis of the *MAX* gene revealed the presence of a novel heterozygous variant, c299G>C (p.Arg100Pro, NM_002382.5) that the bioinformatics prediction programs defined as noxious and causative of pathology.

**Conclusion:** This report represents the first description of a co-occurrence of multiple composite PCC-GN and neuroblastic tumors. The long timeline of the presentation of the NENs/neuroblastic tumors from infancy to adulthood requires a lifelong follow-up for this patient. Moreover, the importance of this case lies in the presence of a novel *MAX* gene variant deleterious, harmful, and causative of pathology, confirmed by Sanger sequencing and never been associated before with multiple composite PCC-GN. The present case underlines the importance of precision medicine and molecular diagnoses for hereditary pheochromocytomas and paragangliomas, suggesting that when they occur in early childhood, it is necessary to perform an extensive genetic investigation and a lifelong follow-up.

## Introduction

Pheochromocytomas (PCCs) are chromaffin tumors arising from the adrenal medulla, while extra-adrenal chromaffin and non-chromaffin paraganglial tumors are classified as paragangliomas (PGLs) (1). PCCs, PGLs, ganglioneuroblastomas (GNBs), and ganglioneuromas (GNs) are neuroendocrine neoplasms (NENs) (1). The International Neuroblastoma Pathology Classification divides neuroblastic tumors into four categories on the basis of their morphology, clinical features, and behavior: neuroblastoma, nodular GNB, intermixed GNB, and GN (2). Neuroblastoma is a malignant tumor comprising neuroblasts with a poor Schwannian stroma, and has the lowest degree of cell maturation and differentiation. It typically undergoes spontaneous regression or differentiation into GNB and GN (3). GNB consists of ganglion cells and neuroblasts with a varying proportion of Schwannian stroma and has an intermediate malignant potential; the nodular subtype is more aggressive, has a worst prognosis and a poorer response to therapy than the intermixed subtype. GN is a benign tumor characterized by the dominance of Schwannian stroma over neuronal elements (4, 5). PCCs are rarely associated with other types of neuroblastic non-PCC tumors with the same embryologic origin, the so-called “composite pheochromocytoma” (6, 7).

From the perspective of precision medicine (8), the recommended workup for NENs includes plasma or 24-h urine fractioned metanephrine, chest/abdominal multiphasic computed tomography (CT), and magnetic resonance imaging (MRI) or fluorine-18-fluorodeoxyglucose (FDG) positron emission tomography (PET) in association with functional diagnostic tests. MIBG scintigraphy and somatostatin receptor scintigraphy as well as Gallium-68 [68Ga] SSA radiotracer PET/TC are valuable techniques especially in cases of multiple tumors and disseminated disease (9, 10). Genetic counseling and genetic testing for a number of genes involved in the pathogenesis of PCCs (*NF1, RET, von Hippel-Lindau gene [VHL], SDHD, SDHC, SDHB, EGLN1/PHD2, KIF1, SDHA, IDH1, FH, HIF2, SDHAF2*, and *SDHAF3*) are recommended when appropriate (11–15). If multiple or CT-negative tumors are suspected, a ^131^I-metaiodobenzylguanidine (MIBG) scintigraphy scan should be performed (16–19).

Alpha blockade (phenoxybenzamine or doxazosin) with aggressive volume repletion associated with preoperative rapid-acting intravenous alpha-adrenergic antagonists and beta-blockers are the mainstay of treatment (20). Tumor excision represents the therapy of choice of non-metastatic PCC/PGL. Whenever possible with metastatic disease, primary tumor resection should be recommended in order to alleviate cardiovascular and other symptoms from catecholamine excess or from tumor invasion. For metastatic PCCs/PGLs, there are few established molecular targeted therapies, which have or may have a positive impact. [68Ga] PET/TC gives the predictive power for the efficacy of peptide receptor radionuclide therapy (PRRT) which, togheter with 131I-MIBG, may have a benificial efficacy in unresectable disease (10, 21). Radiofrequency ablation, cryoablation, and ethanol injection may be considered in the treatment of metastatic (oligo-metastatic) PCC/PGL (22, 23). Conventional chemotherapy (Averbuch scheme and temozolomide) have been widely used (24, 25).

Conventional external beam radiation therapy (cEBRT) or radiotherapy/radiosurgery (gamma-knife/cyberknife) is recommended for locally unresectable disease (18, 19), in the case of bone metastases and may play a significant palliative role in oligo-metastatic disease.

In recent years, growing interest in precision medicine and molecular diagnosis concerning the expanding etiology for hereditary PCCs and PGL has led to the inclusion of *SDHA, TMEM127, MAX*, and *SDHAF2* as susceptibility genes (26–28). Genetic testing is recommended in patients at clinically high risk who are negative for the classic gene mutations (10, 28).

We describe herein the case of a 40-years-old woman with multiple NENs, comprising a pelvic GNB, right adrenal composite PCC-GN, inter-aortocaval PGL, and multiple left adrenal PCCs, associated with a novel variant of *MAX* gene mutation.

## Case Report

Informed consent was obtained from the patient for the collection, analysis, and publication of personal, familial, clinical, and genetic data and the conduct of genetic blood tests. This manuscript was written in accordance with CARE guidelines.

### Pedigree and Family History

**Figure 1:**
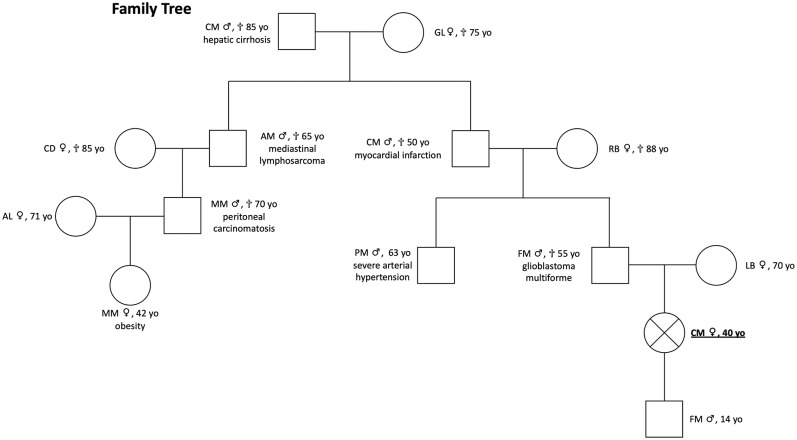
Patient pedigree [proband positive for *MAX* mutation c299G>C (p.Arg100Pro, NM_00.42382)]. She presented clinical signs of multiple PGL and composite PCCs; proband's father had a glioblastoma multiforme, and genetic testing was not performed; proband's son is negative for clinical manifestations, and genetic analyses were not performed.

The patient's pedigree is shown in Figure 1. The proband is a 40-years-old Italian woman of Caucasian ancestry. Her father died from glioblastoma multiforme aged 55 years. She has no siblings. The medical history of her children was negative for any reported disease or apparent NEN-associated clinical signs. Her son (age 14 years) is negative for clinical manifestations and genetic analyses were not performed.

### Case Presentation

**Figure 2:**
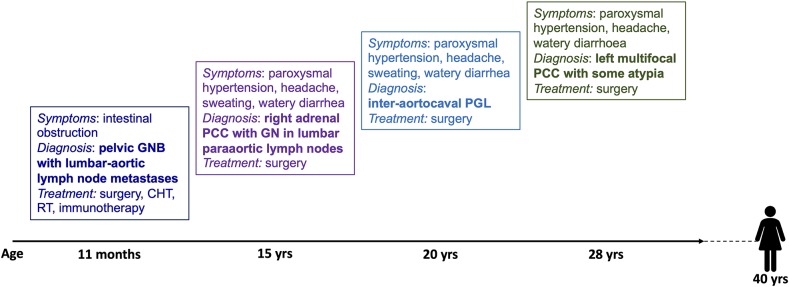
Timing of the presentation of multiple and composite NEN-neural tumors in this patient. GNB, ganglioneuroblastoma; CHT, chemotherapy; RT, radiotherapy; PCC, pheochromocytoma; GN, ganglioneuroma; PGL, paraganglioma.

The timing of the multiple and composite NEN-neuroblastic tumor presentation of the present case is shown in Figure 2.

A 15-years-old Caucasian girl with a history of blood and mucus-free watery diarrhea (up to four times in 24 h), headache, and sweating was referred in 1994 for paroxysmal hypertension. Her personal history included, at the age of 11 months, a GNB with lumbo-aortic lymph node metastases, which was surgically excised and then treated with chemotherapy, radiotherapy, and immunotherapy. Given the symptomatology, an endocrinological workup was performed showing normal 24-h urine vanillylmandelic acid (VMA) and total metanephrines. An abdominal ultrasound and adrenal washout CT scan demonstrated a 5.0-cm right adrenal lesion. The patient underwent abdominal surgery. The histological diagnosis was right composite adrenal PCC-GN. This diagnosis derives from the histological description of an alveolar pattern of round polygonal cells with an eosinophilic granular cytoplasm and spindle cells with a fasciculation pattern that were outlined by collagen fiber septa. The tumor cells showed round, pleomorphic nuclei with prominent nucleoli. The lymph nodes contained ganglioneuromatous tissue. After surgery, the symptomatology soon improved.

**Figure 3:**
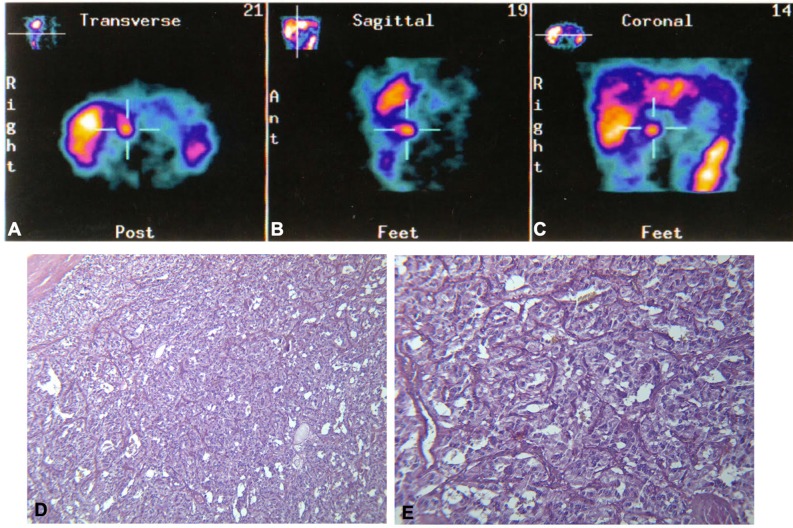
Top: ^131^I-metaiodobenzylguanidine (MIBG) scintigraphy distinctly shows a hot spot in the inter-aortocaval region, as seen in the **(A)** transverse, **(B)** sagittal, and **(C)** coronal scans. Bottom: Histological appearance of the paraganglioma, cords and nests of cellular elements with abundant granular basophilic cytoplasm and ovoid nuclei with prominent nucleoli. **(D)** EE 10×. **(E)** EE 20×.

Five years later, the patient presented with a recurrence of paroxysmal hypertension. Neuroendocrine tumor marker tests were positive for 24-h urinary total metanephrines (2,168 μg/24 h; reference range <354 μg/24 h), 24-h urinary catecholamines (239 μg/24 h; reference range <100 μg/24 h), and VMA (VMA urinary spot test: positive). Serum chromogranin A was also positive (CgA 248 ng/ml; reference range <90 ng/ml), while CEA (1.20 ng/ml; reference range <5 ng/ml), NSE (4.00 ng/ml; reference range <10 ng/ml), and alfa fetoprotein (alfaFP 4.90 ng/ml; reference range <5 ng/ml) were normal. An abdominal CT scan showed a 2.2-cm inter-aortocaval right mass. MIBG scintigraphy showed a distinct hot spot in that region (Figure 3). The patient was therefore referred for surgical treatment. The histological diagnosis was inter-aortocaval PGL (see Figure 3). Histological description showed that the tumor consisted of a trabecular-nesting cell pattern with abundant basophilic granular cytoplasm. The tumor cells had oval nuclei with prominent nucleoli and were strongly positive for CgA and NSE on immunohistochemistry. A positivity to S100 antibodies was found at immunohistochemistry.

**Figure 4:**
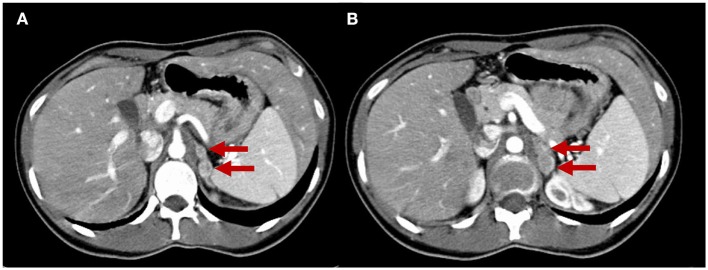
Adrenal CT scan. The two slices **(A,B)** show four nodules in the left adrenal gland measuring from 10 to 18 mm that are unattenuated after contrast administration (red arrows), compatible with left multifocal adrenal pheochromocytomas.

Eight years later, the patient presented with a recurrence of paroxysmal hypertension. The endocrinological workup was positive for 24-h urinary total metanephrines (1,989 μg/24 h), serum noradrenaline (2,100 pg/ml; reference range 300–900 pg/ml), and serum adrenaline (156 pg/ml; reference range 0–100). Serum CgA (260 ng/ml) and alfaFP (5.31 ng/ml) were also positive. CEA (3.00 ng/ml) and NSE (7.70 ng/ml) were normal. Adrenal washout CT scans showed four non-adenomatous left adrenal masses between 10 and 18 mm (Figure 4). Abdominal MRI confirmed these lesions with significantly high signal T2-hyperintensity, suggesting a neuroendocrine (NE) origin. Surgical excision was performed. The diagnosis was *multifocal left PCCs with some atypia*. The macroscopic observation showed four adrenal nodules measured 18, 20, 25, and 27 mm that were enclosed in a thin capsule with a reddish-tan surface and having multiple hemorrhagic areas. Histologically, spindle cells were found in 30% of the neoplasm, with prominent nucleoli. The nodules were strongly positive for CgA and synaptophysin on immunohistochemistry. Large polygonal cells with eosinophilic granular cytoplasm and strong atypia were found in 40% of the nodule tissue. The Ki-67 proliferation index was >5%. There were no sustentacular cells. The symptoms disappeared and plasma and urinary catecholamines and 24-h urinary metanephrines returned to normal levels.

The patient is currently in good health. She is in follow-up with no evidence of metastases and recurrence. The trend of NE markers over the course of the clinical history is shown in Figure S1. A review of the pathology slides of all the resected tumors confirmed the four diagnoses, which can be summarized as metastatic GNB, bilateral metastatic PCCs, and inter-aortocaval PGL.

## Methods

### Genetic Analyses

Next-Generation Sequencing was performed for *VHL, RET, NF1, Tp53, SDHD, SDHB, SDHC, SDHAF2, SDHAF3, SDHA, TMEM127*, and *MAX* genes in the Molecular Diagnostic Laboratory for Hereditary Tumors, Veneto Institute of Oncology, Padova and Genetic Ames Group Laboratory, Naples, Italy. Mutation of the *MAX* gene was confirmed by Integrative Genomics Viewer (IGV), a high-performance visualization tool. Consequently, the *MAX* gene was also analyzed for intragenic mutations by Sanger sequencing.

## Results

No mutations were found in *VHL, RET, NF1, Tp53, SDHD, SDHB, SDHC, SDHAF2, SDHAF3, SDHA*, or *TMEM127*. In the *MAX* gene, a novel heterozygous variant, c299G>C (p.Arg100Pro, NM_002382.5), was found. This variant of germline mutation (from peripheral blood) was analyzed with the bioinformatics prediction programs “Sift,” “PolyPhen,” and “Mutation Taster” at Genetic Ames Group Laboratory, Naples, Italy. See Table 1. The programs have defined that this variant is deleterious, harmful, and causative of pathology. For diagnostic completeness, this variant has been confirmed by Sanger sequencing. The variant is not currently reported in the ClinVAr and dbSNP databases and in GnomAD database. See Figure S2.

**Table 1:** *In silico* prediction table.

**Genetic variant in *Max* gene**	**Predictor tool**	***In silico* predictions information**	**Score**	**Range**
*c.299G>C* *p.Arg100P* *NM_002332.4*	Sift	Deleterious	0.01	Values <0.05 usually considered intolerant
	Polyphen	Probably damaging	0.93	Values more than 0.5 usually considered damaging
**Genetic variant in** ***Max*** **gene**	**Predictor tool**	***In silico*** **predictions information**	**Accuracy**	**Range**
c.299G>C p.Arg100P NM_002332.4	Mutation taster	Disease causing	1	Values close to 1 corresponding to the most “secure” prediction

## Discussion

The lesson from this case report highlights several important messages.

First, the metachronic occurrence of PCCs/PGL and neuroblastic tumors. Although previously PCCs, PGLs, and neuroblastic tumors were thought to share the same embryonic origin from neural crest cells, several recent studies showed that the progenitor cells have partly overlapping origin and that chromaffin cells of adrenal medulla arise from peripheral glial stem cells or Schwann cell precursors (29). The simultaneous presence of PCCs and PGLs is rarely found and it is reported in *RET*-positive patients with MEN2B and also in *SDH* mutated patients (30).

To our knowledge, 34 composite PCC-GN cases are reported in literature (6, 31–45). Most of these did not involve any genetic factors, while four cases involved an association with MEN2 (31, 42), VHL (40), or NF1 (43) syndrome.

In this view, the second message of scientific interest is represented by the association of the new described *MAX* gene heterozygous variant, c299G>C (p.Arg100Pro, NM_002382.5), with the tumors.

An association between a *MAX* gene mutation and composite PCC with a ganglioneuromatous component has never been described in the literature, while just one composite case has been described involving PGL, in an adolescent who had been successfully treated for a stage IV-S neuroblastoma 15 years earlier (46).

*MAX* is a crucial component of the MYC-MAX-MXD1 transcription factor network, which regulates cell proliferation and differentiation and apoptosis (47). The formation of MAX-MXD1 heterodimers counteracts the dimerization of MYC with MAX, which would act as a transcriptional activator (47). *MAX* is thus a tumor suppressor gene and its mutation favors the development of hereditary PCCs and PGLs (28, 46–48).

Germline mutations affecting the MYC-associated protein X (*MAX*) gene are considered a major genetic predisposition factor for the development of hereditary PCC and/or PGL (49). The only tumor suppressor genes known to show a “parent-of-origin” phenotype are the recently described genes *SDHAF2* and *MAX*, located on chromosome 11q12.2 and 14q23, respectively (50).

*MAX* mutations cause hereditary and sporadic PCC/PGL. Genotype–phenotype associations suggested that *MAX* mutations were associated with bilateral PCC and with an apparent paternal transmission of the disease (47).

Little is known about genetic alterations in sporadic tumors. Mutations in PCC/PGL susceptibility genes are detrimental for neuronal precursor cells. This could explain the apparent rarity of somatic mutations in these genes in apparently sporadic PCC/PGL (51).

Third, the genetic pattern is confirmed by the intermediate biochemical phenotype that in NENs' patients seems to be associated with a germline *MAX* gene mutation (52). Given that the patient showed a noradrenalin secretion as biochemical phenotype, “cluster 2” of PCC/PGL would appear to be delineated (10). Indeed, before *MAX* gene identification, our patient had undergone various genetic tests over the years (*RET* for associations with MEN2; *VHL* for associations with Von Hippel Lindau Syndrome; *NF1* for associations with Neurofibromatosis type 1; and *Tp53* for associations with Li Fraumeni Syndrome), all proved negative. Furthermore, *SDH* gene mutation analyses performed to explore PGL context (53) were also negative.

Literature analysis showed that the case of a young woman with adrenergic phenotype and bilateral PCC with PGL associated to a germline mutation in *MAX* gene (c.70_73delAAAC/p.Lys24fs^*^40) was reported by Shibata et al. (54). Recently, a case of a 49-years-old woman with bilateral PCC and adrenergic phenotype plus pituitary prolactinoma was also associated to a pathogenic *MAX* mutation c.296-1G>T (NM_002382), which has not been previously described (55).

Moreover, a 25-years-old patient with multiple PCCs associated with adrenal medullary hyperplasia and with a non-sense germline *MAX* mutation was described (56).

Other data from literature reported a complex *MAX* rearrangement with the loss of the wild-type *MAX* and *FUT8* in a family with malignant PCCs, renal oncocytoma, and erythrocytosis (57).

Given the role of *MAX* as a tumor suppressor gene, the novel heterozygous variant identified and the relative youth of the patient, her follow-up will require considerable attention, given the risk of other endocrine and non-endocrine neoplasms and because loss of *MAX* function is correlated with greater aggressiveness and metastatic potential (47) than the other mutated pathways involved in these types of tumor. The significance of the *MAX* variant (c.299G>C; p.Arg100Pro) is currently unknown (49).

In addition to the multiplicity of tumor lesions, the earliness of onset also represents a peculiar characteristic (46, 58).

The mean onset age among the known described patients was 33 years (range 13–58 years), while in our case, the first presentation was in infancy. This is a crucial point in relation to *MAX* gene penetrance. Indeed, nowadays no reliable penetrance estimations are available for *MAX* mutation carriers.

Our patient had no family history for PCC/PGL. Given that her father died of glioblastoma multiforme, the present study may suggest an evidence of paternal mode of transmission in *MAX* mutation carriers. If this mode of inheritance was confirmed, disease is only passed on to children by their father, resulting in the phenomenon of generation-skipping. In this view, it would be even more important to analyze whether the mutation was transmitted to the patient's son, who would be able to transmit the disease to the offspring as well as the *MAX* genetic mutation (28).

The limitations of this case include the fact that the assessments performed on the patient over the last 25 years are obviously not all in line with the current guidelines. The 24-h urinary VMA measurement is no longer recommended for the diagnosis of PCC (9). For this reason, no clinical significance can be attributed to the normal concentration of 24-h urinary VMA at the time of the second and the third tumor. In fact, a false-negative rate of 41% has been described in PEHO (progressive encephalopathy with edema, arrhythmia and optic atrophy) and neuroblastoma patients (59). In addition, MIBG scintigraphy is less efficacious than FDG- or F-DOPA-PET for the diagnosis of PGLs; however, the latter techniques were not available at the time of the patient's earlier workups.

In conclusion, we describe, for the first time, a novel heterozygous *MAX* gene variant: c299G>C (p.Arg100Pro, NM_00.42382), associated with the occurrence of multiple and composite PCC/neuroblastic tumors, occurred yet from infancy in a young women. The molecular mechanism of the *MAX* gene and the impact of *MAX* mutations require thorough investigation, to enable the prognosis of affected patients to be promptly established and targeted treatments to be developed.

## Ethics Statement

The patient gave her written consent to sample collection, genetic testing, and the use of genetic test data for the purposes of research. The sample collection and preparation protocols were approved by the Department of Experimental Medicine Sapienza University of Rome, Italy. Institutional review board review and approval was not required for this case report. Consent for publication has been obtained from the patient, including permission for the details/images to be available on the Internet and viewable by the general public.

## Author Contributions

EG, CP, and FSe conceived and designed the study. FSe, CD, ES, and RP collected the clinical samples and analyzed and interpreted the patient's data. FSc carried out the genetic analyses that first demonstrated the presence of the *MAX* gene mutation in this patient in 2011. CP, FSe, AF, and EG co-wrote the manuscript. VB, AI, and AL contributed to the revision of the manuscript. All authors have read and approved the final manuscript.

## Conflict of Interest

The authors declare that the research was conducted in the absence of any commercial or financial relationships that could be construed as a potential conflict of interest. The reviewer MP declared a past co-authorship with one of the authors FS to the handling editor.

## Supplementary Material

Figure S1 Trend of NE markers in relation to neoplastic occurrence over the course of the clinical history. CgA, Chromogranin A (<90 ng/mL); U catecholamines, urinary catecholamines (<100 mcg/24 h); U metanephrines, urinary metanephrines (<354 mcg/24 h); VMA, vanillylmandelic acid (urinary VMA spot test: positivity was expressed graphically as a value of 100). The endocrinological work-up in 2008 included serum assessment of noradrenaline and adrenaline, which were not expressed in the panel, while CgA, VMA and U metanephrines were reported.

Figure S2 Sequencing of the *MAX* gene. Sequences of *MAX* mutation in genomic DNA from a venous blood sample. **(A)** shows Sanger image of *MAX* mutation analysis; **(B)** shows the *MAX* mutation analyzed with the Integrative Genomics Viewer (IGV).

## Abbreviations

alfaFP: alfa fetoprotein
CgA: chromogranin A
CT: computed tomography
FDG: fluorine-18-fluorodeoxyglucose
GN: ganglioneuroma
GNB: ganglioneuroblastoma
MIBG: 131I-metaiodobenzylguanidine
MRI: magnetic resonance imaging
NEN: neuroendocrine neoplasm
PET: positron emission tomography
PCC, pheochromocytoma, PGL, paragangliomas. VMA: 24-h urine vanillylmandelic acid.
