# Supplementary figures and images for: A Novel MAX Gene Mutation Variant in a Patient With Multiple and “Composite” Neuroendocrine–Neuroblastic Tumors
Source: Front Endocrinol (Lausanne). 2020 May 19;11:234. doi: 10.3389/fendo.2020.00234 (PMC7249266; doi:10.3389/fendo.2020.00234)

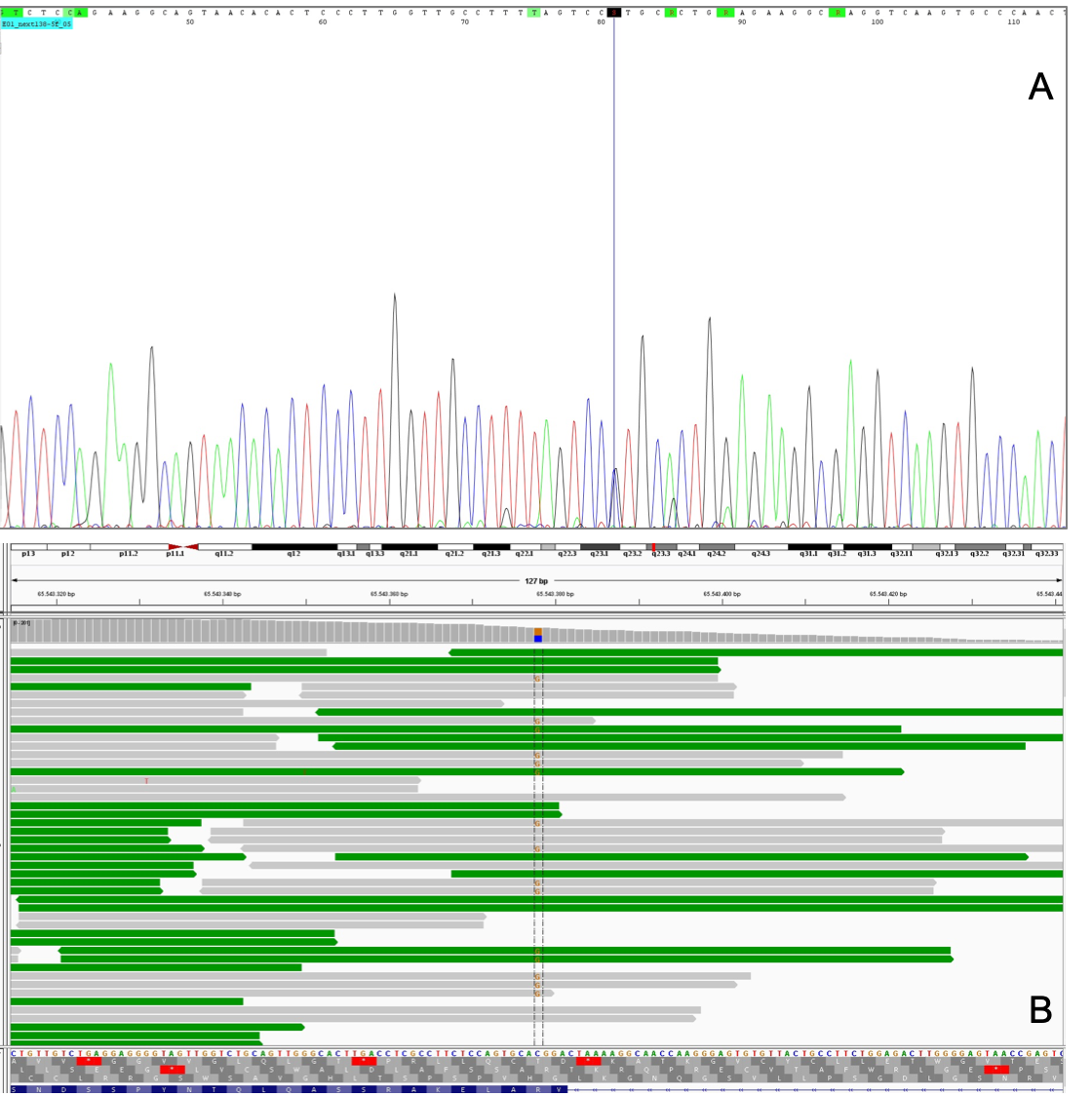

Supplement: Figure S2 — Sequencing of the MAX gene. Sequences of MAX mutation in genomic DNA from a venous blood sample. (A) shows Sanger image of MAX mutation analysis; (B) shows the MAX mutation analyzed with the Integrative Genomics Viewer (IGV). [file Image_2.PNG]
